# Haptic Signals as a Communication Tool Between Handlers and Dogs: Review of a New Field

**DOI:** 10.3390/ani16020323

**Published:** 2026-01-21

**Authors:** Hillary Jean-Joseph, Dalila Bovet

**Affiliations:** Laboratoire Ethologie Cognition Développement (LECD), Université Paris Nanterre, 92000 Nanterre, France

**Keywords:** human–dog, remote communication, interaction, vibrotactile, haptic, harness, vest, animal–computer interaction

## Abstract

Developing new haptic communication tools to enhance communication between dogs and their handlers during field operations has garnered interest in recent years. It is a promising field that could ameliorate dog–handler interactions in the field while addressing practical challenges, such as the need for discrete communication during operations. When extended to the public, such technology could improve communication with impaired dogs. With this review, we aim to (1) give an overview of dogs’ understanding and discrimination of haptic signals, (2) highlight the need to investigate the possible impact of such tools on dogs’ welfare, as well as (3) point out current caveats and future research directions.

## Simple Summary

In recent years, there has been growing interest in developing tools that enable handlers to communicate with their dogs via vibration, a technique known as “haptic communication”. This technology has the potential to enhance handler–dog collaboration, particularly in situations requiring silent or non-verbal communication, and may also improve interactions with sensory-impaired companion dogs. In this review, we examine dogs’ ability to perceive and discriminate vibration-based signals, emphasise the importance of understanding the effects of these tools on canine welfare, and identify future directions for research in this emerging field.

## 1. Introduction

### 1.1. Background

Dogs (*Canis lupus familiaris*) are the first species to have been domesticated [1]. Genetic and archaeological evidence indicate that dogs started to diverge from wolves (*Canis lupus*) around 35,000 years ago [2,3,4] and have shared humans’ environment since then. Hence, dogs and humans share a long common history, which has made dogs perfectly adapted to human society [5]. Thanks to the domestication process and a life alongside humans [6], dogs have developed exceptional socio-cognitive abilities [7,8,9] and an excellent capacity to interpret human communicative cues, including gaze direction, gestures, and emotional expressions [10,11,12,13], and to cooperate with them [14,15]. Nowadays, dogs fulfil different functions in humans’ everyday lives [16] such as hunting partners, livestock guardians, military working dogs (MWDs), assistance dogs, and companions.

Communication refers to the process by which one individual (the sender) produces a signal that influences the behaviour or internal state of another individual (the receiver) [17]. This process requires both the intentional or evolved production of a signal and its effective perception by the receiver [18]. In animal behaviour, communication can occur across multiple sensory modalities—including visual, auditory, chemical, and tactile channels—and serves functions ranging from coordination and bonding to signalling danger or social intent [18]. Dogs’ intra-specific communication uses most of those channels [19], and they also apply them to understand humans’ signals. For example, dogs are skilful in interpreting human emotions via olfactory cues [20] and facial expressions [13,21]. They are also very attentive to humans’ other communicative cues, such as gestures and gaze direction [22], or other ostensive signals [23,24]. Additionally, they are attuned to human attentional states [25,26]. Hence, although the processes differ from those involved in human communication, dogs are able to understand human vocal signals [27].

To communicate with dogs, humans mostly use audio and visual channels, such as verbal commands and audio cues (e.g., clicker training) or various gestures to indicate to dogs what is expected of them. Nevertheless, traditional communication channels, particularly verbal and visual, are vulnerable to environmental interference, including noise, distance, poor visibility, or obstructed lines of sight. In addition, verbal communication and audio cues lack discretion, which is an essential factor during military manoeuvres. In operational contexts such as disaster zones or tactical missions, these constraints necessitate alternative, non-visual and non-auditory communication methods that remain effective under field conditions.

One way to negate the constraint of traditional communication channels is animal–computer interaction (ACI). It is an interdisciplinary field that extends principles from human–computer interaction (HCI) to nonhuman users, integrating insights from animal behaviour, welfare science, and cognitive psychology [28].

Among animal species, dogs are the primary focus of ACI research due to their social intelligence, trainability, and close working relationships with humans [29,30]. Applications range from enrichment tools and welfare monitoring systems to advanced wearable devices that enable bidirectional communication [31]. Within this field, haptic or vibrotactile interfaces have gained attention as a modality particularly well suited to sending messages to trained canines. Indeed, haptic (tactile) communication is the delivery of mechanical cues (e.g., vibration, pressure, or motion) to the body. It has been used in human–machine interaction for decades [32] and is now applied in human–animal interfaces.

Since haptic signals bypass the auditory and visual channels, they allow the reliable and silent (to human ears at least) transmission of information directly through touch. Such communication devices are particularly interesting in the context of working dogs, especially search and rescue (SAR) and military working dogs (MWDs) [30,33]. In fact, the success of canine teams largely depends on the quality of communication between handlers and dogs. Handlers typically give commands to their dogs using their voice or gestures, which requires close proximity to maintain communication during operations. This requires sustained mutual attention in an environment that can be excessively noisy and distracting. Hence, professional canine teams have an urgent need for new tools that can both expand their range of communication and make communication more discreet. The other constraint faced by canine teams is the dogs’ availability and their compatibility with their handlers. Often, a single dog is assigned to several handlers, which can affect the effectiveness of the dog–handler pair. Using a haptic device as a neutral communication modality could help address these issues [34].

Dogs have a rich repertoire of sensory capabilities; olfaction is dominant, but tactile sense is also important—for social touch (e.g., mouthing, pawing), body-contact, vibrations (ground vibrations, human footsteps), and proprioception [19]. Although much less studied than human tactile perception [35], dogs are able to sense vibrations and mechanical cues. The idea of delivering discrete haptic signals to a dog assumes that the animal can reliably perceive and differentiate targeted tactile stimuli (e.g., vibration on different body locations or durations/pulses) through the skin’s mechanoreceptors. In dogs, the challenge lies in delivering consistent tactile energy through fur, variable skin thickness, and diverse body morphologies. Although the structural and functional properties of canine skin are well known [36,37] and should allow dogs to perceive touch and vibration [30], few studies have quantitatively assessed the intensity and frequency ranges that dogs can perceive and discriminate.

### 1.2. Objectives

To our knowledge, no existing review focuses exclusively on wearable haptic communicative devices for communication between humans and dogs. However, one review addresses the use of technology in remote human–dog interaction at large [31] and two others discuss the framework of canine–computer interaction [30,33]. Therefore, this review aims to examine the existing literature on wearable devices designed to enhance human–dog communication through vibrotactile signals, as this emerging field would benefit from a comprehensive overview of the work conducted so far.

## 2. Methods

To ensure that our review was conducted systematically and without bias, we conducted a scoping review using the PRISMA (Preferred Reporting Items for Systematic reviews and Meta-Analyses) methodology [38]. This study was not registered in PROSPERO, as it does not relate to human health.

### 2.1. Eligibility Criteria

The studies included in this scoping review were English-language research articles published in journals and conference proceedings that (1) described wearable haptic technology, (2) aimed at supporting remote human–dog communication, and (3) included testing of the wearable device. Studies that did not fulfil any of these criteria were excluded.

#### 2.1.1. Information Sources

The databases used for this review were SpringerLink, IEEE Xplore, PubMed, BASE, and ScienceDirect. These five databases were chosen because they are recognised as reliable sources of high-quality publications in computer science, technology, engineering, and animal behaviour. The search also included hand-searched papers that were cited in the retrieved articles, as well as reviews on closely related topics.

#### 2.1.2. Search

The specific syntax of the queries varied depending on the database. However, the concepts of (1) wearable haptic devices and (2) human–dog communication and interaction were consistently expressed using the same keywords. The following queries were used in each database:

- (“dog”) AND (“vibrotactile”) AND (“vest”);
- (“dog”) AND (“haptic”) AND (“harness”);
- (“dog-human”) AND (“remote”) AND (“interaction”);
- (“human–dog”) AND (“remote”) AND (“interaction”);
- (“haptic”) AND (“communication”) AND (“dog”).

#### 2.1.3. Study Selection

The screening process was carried out in stages. First, the titles of each result were screened. If the title referred to both dogs and haptic devices, the abstract was reviewed to confirm that the topic addressed remote communication from humans to dogs (and not only from dogs to humans) via wearable haptic devices. Next, the full texts of the selected articles were examined for final inclusion. During this process, duplicate articles retrieved from different databases were identified and removed. Conference papers were included in the reviews if they presented data. The searches were last run on 13 January 2026.

#### 2.1.4. Data Charting and Result Synthesis

The review included all studies that met the inclusion criteria, and the data extracted from these studies were used to address the five research questions:

- Features of the wearable device (e.g., weight, number of actuators, type of actuators);
- Features of the haptic signals (e.g., frequency, acceleration, duration);
- Number of different haptic signals used;
- Number of dogs tested, their breed, and previous training experience;
- Results of the testing (detection threshold and accuracy);
- Consideration of dogs’ welfare during the process (training method used, ethical approval, behavioural observations).

To extract and summarise this data, we conducted a systematic examination of the selected articles and entered all targeted variables into a standardised Excel data matrix. The major findings that emerged from these studies are described below.

### 2.2. Use of Generative Artificial Intelligence (GenAI)

We used ChatGPT (GPT-4-Turbo, OpenAI; model version gpt-4-turbo-2024-04-09) to generate the simple summary for a lay audience.

## 3. Results

### 3.1. Overview

Our search yielded 20,756 records. Queries across five digital libraries identified 20,753 records, with three additional papers found via hand-searching. SpringerLink and ScienceDirect contributed 40% (8311/20,753) and 59.4% (12,325/20,753) of the records, respectively, with other databases accounting for 0.6% (117/20,753). The high number of retrieved records was mainly due to frequent hits for the terms “communication” and “interaction”.

**Figure 1:**
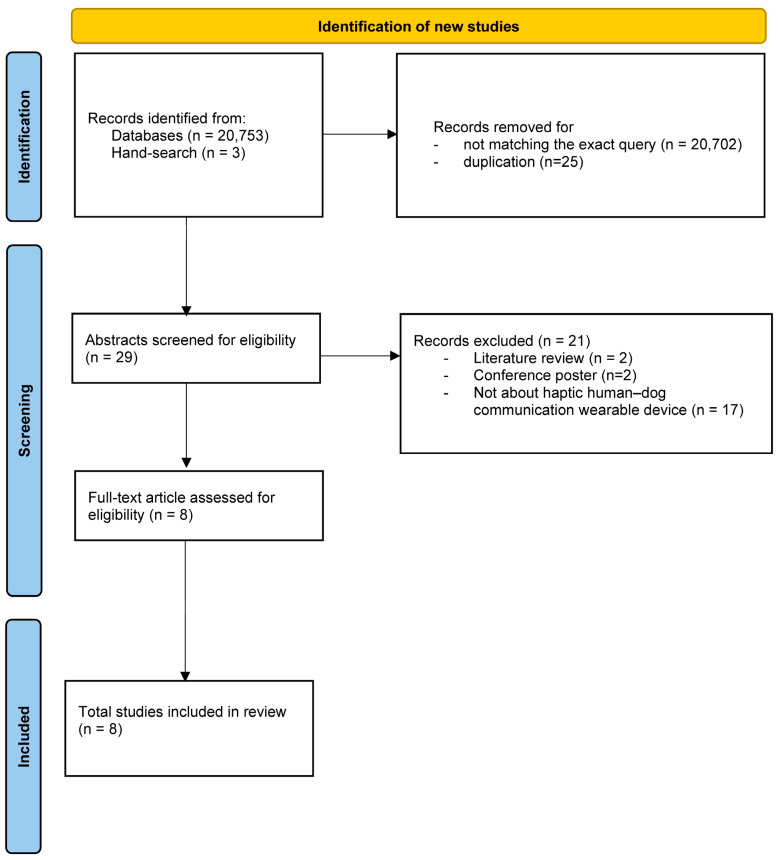
PRISMA [38] flow diagram of the review selection process.

After removing non-matching records (20,702) and all duplicated records (25), we screened 29 papers for eligibility. Based on the exclusion criteria, 72.4% (21/29) of the records were discarded after reading the titles and abstracts. As a result, eight studies were included in this review. Figure 1 shows the flow diagram for the several stages of the review. Table 1 summarises the data extracted from the selected scientific articles.

### 3.2. Studies Reporting Haptic Vest Use Without Numerical Data

Two studies [39,40] reported an embedded sensor vest that monitors a dog’s position, motion, and orientation in real-time and delivers tones and vibrotactile signals to guide the dog. The authors conducted a case study to demonstrate how the system operates in practice. However, both papers focused on the sensors, the navigation system, and the algorithm needed to make the system fully autonomous. Haptic commands were mentioned as a means to guide the dog, but details of the signal features were not provided, aside from the actuator’s placement on the vest. Mostly, they used vibrations to indicate when the dog should go right (a vibration on the front of the right shoulder, indicated as “Vest interior; Front right; Shoulder” in [40]) or left (a vibration on the left side of the back of the ribcage indicated as “Vest interior; Back left; Ribs” in [40]). In addition, neither article presented the data on the dog’s accuracy, but both confirmed that the dog could be guided with vibrations and tones.

### 3.3. Device Features

The base used to create haptic devices varied. Devices range from custom-made harnesses [39,43] to modified commercially available vests [34,44]. The choice of actuator support is critical, as it constrains both the number and placement of actuators. For instance, the Julius-K9 harness (JULIUS-K9^®^, Szigetszentmiklós, Hungary) restricts actuators to a small portion of the dog’s upper back and limits shoulder mobility during movement [46,47]. This is not the case with a Thundershirt^®^ (Thundershirt, LLC, Durham, NC, USA) for example. Across the studies reviewed, the number of actuators ranged from 1 to 4, affecting both signal complexity and stimulation coverage. Another important factor reported is the type of actuator used. There are two main types of technology used in the studies: the Linear Resonant Actuators and the Eccentric Rotating Masses (ERMs). The weight of the devices also differed, with lighter designs (0.44 kg in Golan et al. [34,44]) compared to heavier ones (3.2 kg in Britt et al. [39,40]). However, the weight of the devices was not always clearly reported [41,42,45], an oversight that should be avoided in future work as it is a point of concern for the welfare of the dogs wearing the device. As a rule of thumb, device weight should generally not exceed 10% of a dog’s body mass, though the safe maximum load varies with breed, task, and duration [48]. Consequently, both the harness design and device weight must be tailored to the dog’s breed, training, and operational role. A harness designed for military and working dogs should be adapted for field use, which means it must be as lightweight as possible to reduce fatigue and streamlined to prevent any electronic components from snagging on obstacles.

### 3.4. Signal Features

Acceleration levels and signal duration varied significantly, from 0.3 g to over 5 g, showing wide diversity in intensity. The number of different signals ranged from 1 to 4, and the signal length is often unspecified, except in the work of Golan et al. [34,44] (1.5 s) and Morrison et al. [43] (0.32 s).

The frequency of the signal is another feature of haptic devices that was rarely reported. Often, only the characteristics of the actuators and the batteries were mentioned, but not the haptic signal frequency. However, frequency could be a key factor in designing signals perceptible by dogs but discreet to humans, as frequency affects the sounds humans perceive.

### 3.5. Population Features

The sample size used in the studies presented in this review was rather low, ranging from 1 to 11, with a mean of 2.5 and a median of 1. Most studies used hunting breeds, except for Byrne and colleagues (2017) [42], who used various dog breeds, from miniature Poodles and Papillons to German Shepherds and Border Collies. Dogs’ previous training varied as well; one dog was trained to detect explosives [39,40] and some were trained SAR dogs or trained for agility [42] and hunting [34,43,44].

### 3.6. Dogs’ Perception and Discrimination of the Haptic Signals

The heterogeneity of methods used to evaluate dogs’ success and accuracy across studies hinders direct comparison of findings. Consequently, additional research is needed to establish the scope and sensitivity of canine haptic perception.

In this review, we defined a response as any behaviour a dog presents after receiving a haptic signal. This communication is a success if the dog performed the right behaviour after receiving the haptic signal, i.e., the signal that was associated during training with this specific haptic signal. The accuracy of the dog is the percentage of success; it can be general (i.e., calculated from every haptic signal sent to the dog) or specific to a command.

Byrne and colleagues [42] calculated two variables, the dog’s response rate (DDR) and the dog’s response accuracy (DRA).

$$DDR=\frac{N-D}{N}*100$$

Here, N = number of haptic commands from operator to dog and D = number of deletions, i.e., the dog did not attempt to respond to the haptic command.

Contrary to what Byrne and colleagues postulated, this metric represents the dogs’ perception of haptic signals rather than their understanding of them. This formula takes into account every response the dog performed correctly or not; however, proper understanding of a signal implies responding correctly to it, which corresponds to the DRA.

$$DRA=\frac{N-D-S-I}{N}*100$$

Here, N = number of haptic commands from operator to dog; D = number of deletions, i.e., the dog did not attempt to respond to the haptic command; S = substitutions, where the dog performs the wrong action (i.e., anything but touching the target); and I = insertions, where the dog performs the action without the haptic cue. At the highest level of vibration (5.34 g), average DDR = 90% and average DRA = 93.3%

Morrison and colleagues [43] determined a “comfort” threshold, above which the dog would show signs of discomfort, at 1.1 g and 100 Hz. This threshold was established indoors. The second dog, tested outdoors, did not react to this amplitude and frequency, so the parameters were increased to 2.4 g and 220 Hz. For both dogs, the length of the signal was 320 ms. However, the authors did not provide details on the accuracy calculation, the number of cues given to the dogs, or information on the length of the training. But, they mentioned that during training, one dog showed improvement with a 31% accuracy in the last two sessions with a signal at 2.4 g.

Golan and colleagues [34,44] first measured the perception threshold of their dog subject (0.69 g) and then compared the dog’s responses to vocal and haptic commands. For both vocal and haptic commands dog’s responses were classified in four categories:

- Hit or true positive (HIT), i.e., the dog performed as expected;
- False alarm or false positive (FA), i.e., the dog performed without a command;
- Correct rejection or true negative (CR), i.e., the dog did not receive a command and did not perform;
- Miss or false negative (MISS), i.e., the dog did not perform while receiving a command.

Then they calculated the True Positive Ratio (TPR), a False Positive Ratio (FPR), as well as a Positive Likelihood Ratio (PLR).

$$TPR=\frac{HIT}{HIT+MISS}$$

$$FPR=\frac{FA}{FA+CR}$$

$$PLR=\frac{TPR}{FPR}$$

The results reported were TPR = 0.833 for the vocal commands and TPR = 0.933 for the haptic commands. In both cases, the subject answered correctly (i.e., HIT + CR) 25/30 (83.33%).

Secondly, using the same classification, the authors compared the dog’s ability to discriminate the spatial location of two haptic commands. Finally, they compared the dog’s ability to discriminate the temporal pattern of two haptic signals. They used four different haptic signals, summarised in Table 2.

To assess the dog’s ability to discriminate the spatiality of two haptic commands, they compared the TPR of the “Spin” command before learning the “Down” command (TPR = 0.933) to the TPR after (TPR = 0.866) and found no difference (*p* = 0.439). To assess the dog’s ability to discriminate the temporality of two haptic commands, they compared the TPR of the “Spin” command before learning the “Backpedal” command (TPR = 0.933) to the TPR after (TPR = 0.867) and found no difference (*p* = 0.195). They concluded that dogs can differentiate haptic signals by their spatiality and temporality based on experimental results with four haptic commands.

Hopper and colleagues [45] tested two dogs but presented the quantitative results for only one. This dog was trained to associate three commands with three haptic signals according to Table 3, below.

The response to each haptic command was classified in three categories:

- True positive (TP), i.e., correct behaviour was performed;
- Substitution (S), i.e., incorrect behaviour was performed;
- False negative (FN), i.e., no behaviour was performed.

The perception (P) of the haptic signals was calculated as

$$P=\frac{TP+S}{total\;cues}$$

where “total cues” is the total number of haptic cues sent to the dog.

The accuracy of the spatial discrimination of the dogs was calculated as

$$A=\frac{TP}{total\;behaviours}$$

where “total behaviours” was the total number of behaviours offered in response to a haptic cue (correct or not).

The dog perceived the vibration 76% of the time and was accurate 86% of the time. It is to be noted that the command “Twirl” for which the dog had to turn on his left, was less successful, i.e., had a higher number of FN (the dog did not react) and S (the dog executed another behaviour), which could indicate that this dog was biassed negatively toward its left side. This result is not surprising, as dogs often exhibit a preferred side-bias [49].

The results of these various studies clearly demonstrated that dogs can perceive haptic signals. They can associate commands with various haptic signals and respond accurately to them (using haptic signals alone) after a varying length of training. Additionally, dogs discriminate haptic signals based on the position of the signal on their body (spatial feature of the signal). However, the exact range of their perceptions remains unclear as none of the studies thoroughly tested it.

### 3.7. Dog Welfare

Most studies mentioned ethical approval. Most studies used positive reinforcement to train dogs. Dogs’ behaviour had been monitored during the test in most studies; however, no clear ethogram or behavioural data were presented, making any evaluation of monitoring accuracy impossible. The focus of every article was primarily on the feasibility and technical validation of haptic devices rather than on the behavioural impact of this new technology on dogs.

## 4. Discussion

### 4.1. Main Findings

Taken all together, these articles confirm the following:

- Dogs perceive haptic vibration through their skin;
- Dogs are able, after training, to associate haptic signals with commands;
- Dogs discriminate several features of haptic signals such as intensity (i.e., signal amplitude), spatial location (i.e., where on the body the signals are received), and temporal pattern (i.e., the rhythmicity and continuity of the signal).

Hence, the data suggests that haptic signals can be used to communicate with dogs. However, the results summarised in this review are based on eight scientific articles that employed inconsistent training protocols and experimental designs, as well as very different data analysis methods. Additionally, the number of subjects in each study was rather low, ranging from 1 to 11, with a mean of 2.5 and a median of 1. Therefore, generalising these findings to the entire dog population is not possible. Nonetheless, it is noteworthy that various dog breeds have already been tested and were successful in perceiving haptic signals and associating them with commands [42].

### 4.2. Caveats

The biggest caveat is the lack of consistency in the methods used to train the dogs and to analyse the results. Another issue is the insufficient reporting of important features of the haptic devices, such as the combined weight of the electronic component and the harness, as well as the features of the signals used during the experiment, including amplitude, frequency, duration, and temporal pattern (e.g., whether the signal is continuous or discontinuous, and its rhythmicity).

The absence of this information makes comparisons between different experiments very challenging. It also hinders attempts at replication, which in turn hampers any attempt to build upon past experiments, as new studies must start from scratch, a real hindrance to the further development of the field.

Another pressing issue is that the exact haptic perception range of dogs has never been formally measured. Consequently, researchers currently adjust stimulus intensity based on the reactions of individual dogs during training. The field would benefit from a standardised scale to guide such experiments. Additionally, factors such as dog size, age, coat type, and breed may influence haptic perception and should be considered in future studies.

An additional limitation is the absence of control tests to confirm that dogs rely exclusively on cutaneous perception, rather than auditory cues, to differentiate haptic signals. Even if the signals are supposedly designed to be silent, this silence only concerns the human hearing range and not the dog’s hearing range. In fact, human and dog hearing ranges differ, with dogs exhibiting greater sensitivity to higher frequencies than humans [50]. However, haptic communication refers to the transmission of information through mechanical stimulation of the skin or musculoskeletal system, perceived via tactile and kinaesthetic sensory pathways [51]. Hence, by definition, haptic communication is modality-pure [52], and although vibration motors can produce audible buzzing or clicking, this sound is considered an unintended auditory by-product, and therefore not part of the haptic channel [52]. The presence of audible artefacts may introduce cross-modal contamination, in which the dog responds to sound cues rather than tactile stimulation. For this reason, studies designing wearable communication devices for working dogs should endeavour to minimise acoustic leakage and ensure, thanks to control tests, that the signal remains a pure haptic stimulus. To ensure that dogs rely solely on the haptic channel to perceive the signal, their ears could be temporarily blocked to minimise auditory input. Alternatively, the audio artefact produced by the actuator could be recorded and played back to the dogs without accompanying vibrations. If the dog responds, this would indicate that auditory cues, rather than haptic signals alone, are being used.

### 4.3. Welfare Concerns

While most of the studies mentioned that they monitored dogs for behaviours related to stress, fear or discomfort, and provided a short list of behaviours to watch out for, none contain a proper behavioural repertory (see Table 4) nor a clear end point of the experiment, i.e., a concise listing of the behaviour or reaction the dog could display that would automatically stop the current testing. For example, any attempt by the dogs to escape the harness, either by trying to remove it or escape the room, should warrant a pause in the experiment and a longer habituation protocol. Any sign of fear (involuntary miction or defecation, cowering, etc.) should be a criterion to stop the experiment. Additionally, no quantitative data were provided about the dogs’ behaviours. It remains unclear whether they displayed signs of discomfort and, if so, how many and how frequently. Also, no baseline outside of the test was provided; for example, the number of behaviours when given vocal commands was never observed nor compared to the number of behaviours displayed during a haptic command session. This procedure is essential to verify that interaction with the haptic device does not elicit acute stress in the dog. Furthermore, potential long-term effects should be evaluated by measuring its influence on chronic stress.

The choice of training method is essential for ensuring the dogs’ welfare and should consistently employ non-coercive, positive reinforcement-based approaches [53,54]. In addition, more papers should document the training phase, as it could provide valuable information about how dogs are suited to this type of communication. For example, the length of the training needed to reach a 90% accuracy threshold is valuable data.

As a final point, the health status of the dogs participating in the study was never mentioned. It is, however, important to verify, as health issues such as arthritis could negatively affect a dog’s perception of haptic signals [55]. Additionally, as dogs age, their sensory and cognitive capabilities may decline [56], potentially affecting their perception of haptic signals.

### 4.4. Vibrating Collars and Impaired Dogs

No peer-reviewed studies were found regarding the use of vibrating collars to facilitate communication with blind or deaf dogs; consequently, these devices were excluded from the review. However, this method has been described in several articles pertaining to the care of impaired dogs [57,58]. Owners used either a commercial vibrating collar or an e-collar set to deliver vibration only, without electrical shock, to regain the dog’s attention without relying on visual or auditory cues, as is typical for dogs with normal vision and hearing. We do not support the use of e-collars for dog training, as ample scientific evidence shows that they are less effective than positive reinforcement and detrimental to dogs’ welfare [53,54]. Additionally, their use is legally banned in several countries in Europe (Austria, Denmark, Finland, Iceland, France, Germany, the Netherlands, Norway, Slovenia, Spain, and Sweden).

Compared to a vibrating collar, a haptic harness may be more practical, as it can convey several distinct haptic signals corresponding to commands such as “Heel”, “Sit”, “Down”, or “Stop”, eliminating the need for audio or visual cues.

### 4.5. Future Directions

ACI and even more so Canine-Centred Computing [30] are promising fields with research directions to explore. Concerning the domain of haptic communication toward dogs, several important research gaps remain unaddressed:

- The exact haptic perception range of dogs needs to be evaluated.
- The impact of the different features of the signal on its perception and discrimination needs to be addressed.
- Possible cross-modal contamination needs to be controlled for.
- The ability of dogs to respond to haptic signals after a period with no exposure needs to be evaluated.
- The impact of haptic communication tools on the handler–dog bond needs to be assessed.
- There is a need for greater standardisation of the methods and better reporting of signal features and results to increase experiment repeatability and cross-study comparisons.
- The haptic harness technology needs to be paired with a feedback system that will allow handlers to monitor the dog’s position during operations, including visual feedback.
- The welfare of dogs needs to be better considered, with a thorough evaluation system based on ethological methods.
- Studying human–dog communication mediated by technology is an interdisciplinary topic; therefore, interdisciplinary teams that include ethologists specialised in dog behaviour would greatly benefit this research area.

## 5. Conclusions

Research on haptic communication between humans and dogs is still in its early stages, but the results are promising. Dogs appear capable of perceiving even brief haptic signals and discriminating them based on intensity, spatial location, and temporal pattern. Nonetheless, the field remains nascent and would benefit from greater consistency in terminology and standardisation of procedures, including experimental design, appropriate controls, data analysis methods, and reporting practices.

## Figures and Tables

**Table 1:** Summary of the different features in the reviewed articles.

Article	Device Features			
	Wearable Type	Nb Actuators	Actuator Type	Weight (kg)
Britt et al. 2011 ^1^ [39] Miller et al. 2012 ^1^ [40]	Custom made	2	Not reported	3.2
Byrne et al. 2014 [41]	Modified ThunderShirt	2	Not reported	Not reported
Byrne et al. 2017 [42]	Julius-K9 power harness	1	LRA	Not reported
Morrison et al. 2016 [43]	Customised ThunderShirt	2	ERM	0.75
Golan et al. 2019 ^2^ [34,44]	Commercially available vest	4	ERM	0.44
Hopper et al. 2024 [45]	Julius-K9 power harness	3	ERM	Not reported
**Article**	**Signal Features**			
	**Acceleration (g)**	**Actuators Location**	**Signal Duration (s)**	**Nb Signals**
Britt et al. 2011 ^1^ [39] Miller et al. 2012 ^1^ [40]	Not reported	Left rear Right front shoulder	Not reported	2
Byrne et al. 2014 [41]	Not reported	Left and right sides of ribcage	Not reported	2
Byrne et al. 2017 [42]	4 levels: 0.935 2.694 3.939 5.343	Back of neck	3	4 but only one command
Morrison et al. 2016 [43]	0.3 to 2.4	Right and left above front leg joint	0.32	2
Golan et al. 2019 ^2^ [34,44]	0 to 2.13	Front R and L back R &L	1.5	4
Hopper et al. 2024 [45]	0.8	Middle of the back, right, left side of the ribcage	Not reported	3
**Article**	**Population Features**			
	**Sample Size**	**Breeds**	**Previous Training**	
Britt et al. 2011 ^1^ [39] Miller et al. 2012 ^1^ [40]	1	Labrador Retriever	Hunting Dog, Explosive detection	
Byrne et al. 2014 [41]	1	Border Collie	Not reported	
Byrne et al. 2017 [42]	11	Various breeds	Various training backgrounds	
Morrison et al. 2016 [43]	4	2 Kleiner Münsterländer	Hunting Dogs	
		2 Labrador	Hunting Dogs	
Golan et al. 2019 ^2^ [34,44]	1	Labrador Retriever/German Shepherd cross	Partial Guide Dogs	
Hopper et al. 2024 [45]	2	Border Collie	Operant training	
		Border Collie/Australian Shepherd cross	No specific training	
**Article**	**Main Results**			
	**Detection Threshold**	**Accuracy**	**Main Outcome**	
Britt et al. 2011 ^1^ [39] Miller et al. 2012 ^1^ [40]	Not reported	Not reported	Pilot study demonstrated that a dog can use vibration as a command to turn left or right.	
Byrne et al. 2014 [41]	Not reported	Not reported	A dog can discriminate vibration on its left side from its right side.	
Byrne et al. 2017 [42]	0.935 (8/10 dog)	2.694 (1/10 dog) 3.939 (2/10 dog)	Dogs can be trained to respond to haptic cues. Dogs might be able to differentiate vibration depending on their intensity.	
Morrison et al. 2016 [43]	0.3	Max 31% at 2.4 g	Dogs respond to haptic cues. Prior training can affect those responses.	
Golan et al. 2019 ^2^ [34,44]	0.69	93%	A dog can distinguish between different haptic cues, both spatially and temporally.	
Hopper et al. 2024 [45]	0.8	Variable accuracy	Dog can discriminate between haptic cues’ spatiality but struggle stopping while receiving a haptic cue during a movement without prior training in such exercise.	
**Article**	**Dog Welfare**			
	**Ethical Approval**	**Behavioural Observations**	**Training Method Used**	
Britt et al. 2011 ^1^ [39] Miller et al. 2012 ^1^ [40]	Yes	Dog behaviours mentioned but no data presented	Not reported	
Byrne et al. 2014 [41]	Not reported	Not reported	Not reported	
Byrne et al. 2017 [42]	Yes	Dog behaviours mentioned but no data presented	Operant conditioning and Positive reinforcement	
Morrison et al. 2016 [43]	Yes	Dog behaviours mentioned but no data presented	Positive reinforcement	
Golan et al. 2019 ^2^ [34,44]	Yes	Dog behaviours mentioned but no data presented	Positive reinforcement	
Hopper et al. 2024 [45]	Yes	Dog behaviours mentioned but no data presented	Positive reinforcement	

^1,2^ Describes the same device.

**Table 2:** Command and corresponding haptic cue adapted from the table in [34].

Command	Description	Cue Location	Vibration Type
Spin	Turn around	Front Right	Constant
Down	Lie down	Rear Both	Constant
To me	Approach handler	Front Left	Constant
Backpedal	Walk backward	Front Right	Pulsing

**Table 3:** Command and corresponding haptic cue in [45].

Command	Description	Location Cue
Twirl	A counterclockwise turn	Left
Spin	A clockwise turn	Right
Red	A nose-touch to a red target	Middle

**Table 4:** Non-exhaustive list of behaviours to monitor as they could be stress-related.

Behaviours	Definition
Vocalisations	To whine, whimper, or yelp.
Mouth licking	Tongue moves over the lips.
Panting	To gasp for breath. The tongue is visibly moving in and out of the mouth.
Scratching	To nibble or scratch different body parts with front or hind paws.
Shaking	To wiggle the whole body, starting with the head and finishing with the hind part of the body.
Trembling	The whole body or part of the body is shivering.
Yawning	To open the mouth widely, close the eyes slightly, and move the ears backwards.
Lift paw	One of the front limbs is held up.
Whale eyes	The eyes are wide open, and the sclera is clearly visible.

## Data Availability

No new data were created or analyzed in this study. Data sharing is not applicable to this article.

## Abbreviations

The following abbreviations are used in this manuscript:

ACI	Animal–Computer Interaction
ERM	Eccentric Rotating Mass
HCI	Human–Computer Interaction
LRA	Linear Resonant Actuator
MWD	Military Working Dog
SAR	Search and Rescue
